# Functional Contribution and Targeted Migration of Group-2 Innate Lymphoid Cells in Inflammatory Lung Diseases: Being at the Right Place at the Right Time

**DOI:** 10.3389/fimmu.2021.688879

**Published:** 2021-06-10

**Authors:** Stefan Wirtz, Anja Schulz-Kuhnt, Markus F. Neurath, Imke Atreya

**Affiliations:** ^1^ Department of Medicine 1, University Hospital of Erlangen, Erlangen, Germany; ^2^ Deutsches Zentrum Immuntherapie (DZI), Erlangen, Germany

**Keywords:** Innate lymphoid cells, ILC2, airway inflammation, immune cell trafficking, tissue migration

## Abstract

During the last decade, group-2 innate lymphoid cells (ILC2s) have been discovered and successfully established as crucial mediators of lung allergy, airway inflammation and fibrosis, thus affecting the pathogenesis and clinical course of many respiratory diseases, like for instance asthma, cystic fibrosis and chronic rhinosinusitis. As an important regulatory component in this context, the local pulmonary milieu at inflammatory tissue sites does not only determine the activation status of lung-infiltrating ILC2s, but also influences their motility and migratory behavior. In general, many data collected in recent murine and human studies argued against the former concept of a very strict tissue residency of innate lymphoid cells (ILCs) and instead pointed to a context-dependent homing capacity of peripheral blood ILC precursors and the inflammation-dependent capacity of specific ILC subsets for interorgan trafficking. In this review article, we provide a comprehensive overview of the so far described molecular mechanisms underlying the pulmonary migration of ILC2s and thereby the numeric regulation of local ILC2 pools at inflamed or fibrotic pulmonary tissue sites and discuss their potential to serve as innovative therapeutic targets in the treatment of inflammatory lung diseases.

## Introduction

The lungs are the major organs of the respiratory system in mammals and birds and constantly provide the body with essential amounts of oxygen. Gas exchange in the human lung requires the consumption of enormous amounts of air each day and thus exposes the thin pulmonary mucosal surface with quantities of rather harmless organic and inorganic particulate matter such as aerosols, smoke and pollen (1). At the same time however, there is inevitable exposure to viruses, bacteria and other respiratory pathogens that could compromise proper lung function and overall body wellbeing. As a result, complex and tightly regulated immunological networks evolved to provide both effective tolerance to environmental antigens and protection against potentially invasive airborne pathogenic threats. Many different cell types, such as epithelial cells, myeloid cells and innate and adaptive lymphocytes contribute to cellular and humoral immunity in the lung (2). While the lung already harbors numerous immune cells in the steady-state, their frequencies and relative proportions strongly change during infections, tissue damage or chronic inflammatory diseases. At homeostasis, different subsets of lung resident myeloid cells including bronchial, interstitial and alveolar macrophages as well as dendritic cells comprise the vast majority of pulmonary leucocytes and have been shown to largely support pathogen clearance, tolerance mechanisms and tissue repair (3). However, more recently increasing evidence suggests that innate lymphoid cells (ILCs) substantially contribute to innate immune surveillance of the lung in the steady-state and also play vital roles during physiological or dysregulated chronic inflammatory reactions (4).

ILCs comprise heterogeneous groups of developmentally related lymphocytes that characteristically lack recombination-activating gene (Rag)-dependent rearranged antigen receptors. Mainly based on functional measures, developmental trajectories and transcription factor/cytokine expression patterns, they are broadly categorized into five major subgroups [natural killer (NK) cells, ILC1s, ILC2s, ILC3s, lymphoid tissue-inducer cells (LTi)] that functionally resemble analogous T cell counterparts (5). In both mice and humans, most subsets of ILCs are particularly enriched in tissues harboring barrier functions such as gut, lung and skin and possibly their main biological function is related to their capacity to rapidly respond to danger and stress signals derived from e.g. epithelial or stromal cells. In lungs of naïve mice, ILC2s represent the most frequent ILC population. Upon exposure to alarmin-like proteins released during tissue damage or infections, lung ILC2 numbers rapidly increase and substantial amounts of the type-2 signature cytokines IL-5, IL-9 and IL-13 and also IL-4 are released (6). ILC2 development from common hematopoietic progenitor populations depends on the transcription factors GATA3 and retinoic acid receptor-related orphan nuclear receptor-alpha (RORα). It was shown in parabiosis experiments with genetically distinguishable mice that in the steady-state and during parasitic infections lung ILC2s are largely tissue resident (7) and mostly renew from local pools established in the neonatal period (8). However, as in detail outlined in the following chapters, the extent to which lung ILC2s are strictly tissue resident is currently discussed, as recent studies also suggest that ILC2s can migrate into the lungs from other tissue sites under certain circumstances.

Here, we summarize recent advances in our understanding of the involvement of ILC2s to lung homeostasis and diseases with a particular focus on the immunological aspects that regulate their migratory capacity and thereby their pulmonary accumulation during inflammatory processes.

## ILC2s as Gatekeepers of Lung Homeostasis

### ILC2 Expansion in the Postnatal Period

How ILC progenitors (ILCP) disseminate into peripheral tissues such as throughout embryonic development remains incompletely understood. However, the profound tissue compartmentalization of different ILC subsets suggests that tissue‐specific niches exist that drive the establishment of local ILC pools. Although at low frequencies, ILC2s have been described to populate the human lung already in the second trimester of the fetal period (9). In line, some ILC2s were found in murine embryonic lungs just prior birth. Remarkably, the postnatal window from birth to weaning is characterized by a rapid and pronounced accumulation of ILC2s and other type 2 innate cells such as eosinophils, mast cells and basophils. Because their numbers again decline after weaning and this was not observed in other postnatal organs (10), it was proposed that ILC2s might inhibit overwhelming immune reactions to airborne particles that naïve lungs are exposed to in the context of alveolarization. De Kleer et al. also found that these postnatal lung ILC2s all expressed the IL-33 receptor ST2, proliferated in response to stimulation with IL-33 and produced IL-5 and IL-13. In line with an important role of innate type 2 immunity for lung tissue repair and remodeling, increased production of IL-33 by the lung epithelium was observed (11). Although it is currently unclear which factors trigger neonatal IL-33 production, it is feasible that mechanical and oxidative stress initiated by exposure to air after birth is of primary importance (12). Recent studies using IL-33 reporter mice also confirmed that, in addition to epithelial type 2 pneumocytes, adventitial stromal cells produce IL-33 in lungs of neonatal and adult mice (13). Notably, scRNA-seq analysis of neonatal lung ILC2s revealed the existence of ILC2 subsets with distinct proinflammatory and tissue-repairing functions that potentially depend on the nature of activation signals (14). Taken together, these recent data clearly indicate that the neonatal period is critical for the establishment of pulmonary ILC2 populations and may already prime lung homeostasis and host defenses during adulthood.

### Regulation of Pulmonary ILC2 Activity

It is believed that type 2 responses evolved to monitor damage to barrier surfaces and to contain or eliminate the pathologic agent in a way that minimizes tissue damage and provides rapid resolution of inflammation. As early sentinels of a lost barrier integrity, lung ILC2s are endowed with a large spectrum of cellular receptors enabling rapid and effective detection of micro-environmental changes and intercellular communication with epithelial-, stromal- and hematopoietic cells *via* soluble mediators or direct cellular interactions.

#### Regulation by Cytokines and Chemokines

Characteristically, ILC2s strongly respond to stimulation with the alarmin-like molecules IL-25, IL-33 and thymic stromal lymphopoietin (TSLP), which are mainly released by type 2 pneumocytes, tuft cells and subsets of lung myeloid cells. As a result, ILC2s are able to secrete on a per cell basis large amounts of IL-5, IL-13, IL-9 and IL-4 thereby mediating eosinophil recruitment, alveolar macrophage polarization, mast cell activation, goblet cell mucus production and smooth muscle contraction (15, 16). By promoting ILC2 survival, IL-9 initiates a positive feedback loop that amplifies ILC2 cytokine production (17). While in the steady-state murine lung “natural” ILC2s (nILC2s) were shown to be largely tissue resident and more reactive to stimulation with IL-33 than IL-25, more recent studies identified a circulating “inflammatory” ILC2 (iILC2) subset. These ILC2s are dependent on the AP1 family transcription factor BATF (18) and can transiently accumulate in the lung after mobilization in the gut or bone marrow *via* systemic IL-25 administration or nematode infection (19). Compared to nILC2s, iILC2s were phenotypically characterized by higher expression of the IL-25 receptor chain IL17RB and KLRG1 and lower expression of the IL-33 receptor chain ST2 and the enzyme arginase 1 (Arg1) (18, 20). Recently, a human inflammatory subset with transcriptional and functional similarities to mouse iILC2s was described in inflamed mucosal tissues. These cells expressed CD45RO, were linked to severe atopic diseases and displayed resistance to corticosteroid therapy, thereby representing potential targets for therapeutic intervention (21).

Reflecting the presence of several alternative activation pathways, lung ILC2s are known to be activated by several further soluble mediators in addition to alarmin-like cytokines. While IL-7 is important for their lineage commitment and drives their proliferation, ILC2s express high levels of the IL-2 receptor chain CD25 and IL-2 derived mainly from T cells, but also other cellular sources is vital for lung ILC2 survival and supports their alarmin-dependent production of IL-5 and IL-13 (22). Furthermore, ILC2s express death domain receptor 3 (DR3), a member of the tumor necrosis factor (TNF)-receptor superfamily, which is activated by its cognate ligand TL1A (23). In mice, ILC2 proliferation and activation were increased upon *in vivo* stimulation with TL1A in a DR3-dependent manner. DR3 is also expressed on human ILC2s and TL1A binding was able to induce IL-5 and IL-13 production *in vitro* suggesting that the TL1A/DR3 axis is an important pathway for ILC2 co-stimulation. Notably, mouse and human ILC2s can not only produce copies amounts of IL-4, but also express a functional IL-4 receptor. Consistent with an important role of IL-4 for ILC2 regulation, Motomura et al. demonstrated that basophil-derived IL-4 stimulated their pulmonary cytokine and chemokine production (24). In humans, IL-4 stimulation of lung ILC2s in the presence of alarmins was important for induction of GATA3 and CRTH2 expression and the secretion of IL-5 and IL-13 (25), suggesting that IL-4 could serve as a critical costimulator of ILC2s.

Interestingly, ILC2s display characteristic chemokine receptor expression patterns and were recently shown to be subject of substantial chemokine dependent regulation. In this context, the C-C motif chemokine receptor 8 (CCR8) was shown to be a critical regulator of ILC2s during parasitic infections and allergic lung diseases. Notably, the two known cognate chemokines CCL1 and CCL8 are produced by different cells in the lung and can have divergent functions. ILC2s are an important *in vivo* source of CCL1 and support their proliferation and effector functions *via* an auto-regulatory positive feedback loop (26). By contrast, CCL8 was shown to be primarily produced by inflammatory CD11c^+^ mononuclear phagocytes (27) and controls the local positioning of ILC2s within the lung during type 2-mediated inflammation (28).

ILC2s are subject of substantial negative regulation and several cytokines turned out to be important for the control of overshooting type 2 responses in the lung. For example, ILC2s express receptors for both type I and type II interferons on their surface (29) and their prototypic functions are strongly impaired by type I IFNs and IFN-γ *in vitro* and *in vivo* (30, 31). A further inhibitory mediator is the IL-12-related cytokine IL-27 that suppresses lung ILC2 activation *in vivo via* STAT-dependent signal transduction pathways (32). Interestingly, studies also identified Treg derived IL-10 and TGF-β as strong inhibitor of murine and human ILC2 effector cytokine production (33, 34). Conversely, TGF-β production of pulmonary epithelial cells rather seems to drive ILC2 proliferation and chemoactivity of murine ILC2s (35), indicating that cell type- or context-specific roles of TGF-β may control ILC2 responses.

#### Regulation by Lipid Mediators

A plethora of lipid mediators including prostaglandins and leukotrienes are present in lungs during type 2 inflammatory reactions and there is growing evidence that several of them regulate biological functions of murine and human ILC2s. Xue et al. demonstrated that the prostaglandin D2 (PGD2) binds to CRTH2 on human ILC2s and induced strong production of type 2 cytokines as well as upregulation of the receptor chains of IL-33 and IL-25 (36). These data were later confirmed in mice (37). Notably, more recent findings demonstrated that activated human blood and tonsillar ILC2s produce PGD2 *in vitro* indicating that the PGD2/CRTH2 axis represents an important autocrine/paracrine pathway of ILC2 stimulation (38). Human ILC2s also express receptors for the leukotrienes CysLT1 and CysLT2 and respond to stimulation with leukotriene C4 (LTC4) and leukotriene D4 (LTD4) and potentially other related ligands (39, 40). Other studies indicated that a similar mechanism is functional in lungs of mice and demonstrated that LTC4 signaling promotes nuclear translocation of the transcription factor NFAT (41). Importantly, LTD4 was shown to promote IL-4 secretion by ILC2s more efficiently than the alarmins IL-25 and IL-33 (39, 42). Notably, some lipid mediators such as lipoxin A4, PGI2 and PGE2 were shown to bind to their receptors on ILC2s to inhibit cytokine production (43–45). Overall, these findings indicate that lipid mediators can act in concert with or independent of alarmins to fine tune lung ILC2 responses in specific tissue contexts and represent interesting therapeutic targets to modulate ILC2 functions in atopic diseases.

#### Interactions of ILC2s with the Nervous System

The lung is densely innervated by different sympathetic, parasympathetic and sensory nerve fibers. Given the well-known implication of neuro-immune interactions in several lung inflammatory conditions and the anatomical proximity of ILC2s and neuronal cells (46), many recent studies investigated molecular mechanisms underlying the functional crosstalk of the nervous system with ILC2s. Thereby, a number of receptors for neuronal transmitters including the neuropeptides neuromedin U (NMU) and neuromedin B (NMB), vasoactive intestinal peptide (VIP), calcitonin gene–related peptide (CGRP) and β2 adrenergic receptor (β2AR) activators are expressed in human and mouse ILC2s, suggesting that ILC2s are a central target of the nervous system’s capacity to rapidly control innate type 2 responses. Compared to other immune cells, ILC2s were identified as the immune cell subset possibly expressing the highest levels of the neuromedin U receptor 1 (NMUR1). Indeed, several parallel studies described a strong *in vitro* responsiveness of lung and gut ILC2s to NMU stimulation that was independent of IL-33 co-stimulation. *In vivo* studies with NMUR1 deficient and NMU-treated mice confirmed a role of NMUR signaling in ILC2s in models of parasitic infections and asthma (47–49).

CGRP released from lung neuroendocrine cells also promoted ILC2 activation in combination with IL-33 or IL-25 in lung inflammation induced by ovalbumin (OVA) challenge (50). However, three subsequent reports employed single cell sequencing as well as *in vitro* and *in vivo* approaches to identify CGRP signaling as a negative regulator of at least subsets of ILC2s, indicating that CGRP may control ILC2s in a context-dependent manner (51–53). While the role of CGRP thus seems to be ambiguous, signaling through the β2AR has been shown to negatively regulate ILC2 proliferation and cytokine production. Consistently, mice lacking β2AR were more prone to excessive type 2 mediated lung inflammation, whereas treatment of wildtype mice with an β2AR agonist was associated with impaired ILC2 responses and reduced lung pathology (54).

Moreover, the bombesin-related peptide NMB, a molecule previously implicated in smooth muscle contraction and metabolic processes, was shown to curb ILC2 responses. Interestingly, this effect was related to direct interactions with basophils as their presence was required for upregulation of the NMB receptor on ILC2s (55). Collectively, these studies clearly suggest that signals from peripheral neuronal cells can potentiate or inhibit type 2 immune responses in the lung *via* ILC2s.

### Role of ILC2s in Lung Infectious Diseases

The respiratory tract continuously interacts with the environment and is therefore possibly the body’s most common target for infectious diseases. Indeed, a large spectrum of different pathogens including large metazoan parasites, fungi, viruses and bacteria can infect the lung and cause worldwide millions of deaths per year. ILC2s are sentinels of damage and mediators of tissue repair and are thus critically involved in the pathogen-directed pulmonary immune response.

#### Viral Infections

Already early after initial description of ILC2s, an accumulation of ILC2s in mouse models of influenza virus infection was observed, which most likely is a direct consequence of alarmin release in the context of infection-dependent epithelial disturbance and necrosis. In 2011, Monticelli et al. observed increased lung numbers of ILC2s after influenza infection. Production of the epidermal growth factor ligand amphiregulin (AREG) by ILC2s supported epithelial restoration in this model (56). Conversely, a further study identified activated ILC2s as a driver of virus-induced airway hyperreactivity after infection with H3N1 influenza (57). During resolution of experimental influenza infection, ILC2s were identified as the major cellular source of the cytokine IL-5 thereby inducing a progressive pulmonary accumulation of eosinophils (58). Additional studies identified influenza infection-dependent plasticity of ILC2s towards ILC1-like cells characterized by low expression of Gata3 and upregulated expression of IL-18Rα, IL-12Rβ2 and IFN-γ (59). Importantly, IFN-γ production during viral infections suppressed ILC2s and their tissue protective capacity (59). IL-33-dependent lung ILC2s were also shown to play a role during infections with respiratory syncytial virus (RSV). Although RSV infections typically provoke dominant Th1 type responses that mediate viral clearance, type 2 cytokines expression early in life may support exacerbated disease activity and even susceptibility to asthma development later in life. In RSV-infected neonatal mice, lung IL-33 expression is upregulated and linked to ILC2 accumulation. In line with this, blockade of IL-33 signaling improved lung pathology without altering viral loads (60, 61). Interestingly, anti-IL-33 treatment was more effective in reducing ILC2 numbers in infected neonates, whereas in adults, secretion of TSLP was shown to more prominently activate lung ILC2s (62). A potential role of dysregulated ILC2 activation also seems to contribute to respiratory tract infections with rhinoviruses (RV). Experimental RV infection of neonatal but not adult mice was associated with the development of asthma-like symptoms, which were associated with alarmin-dependent lung accumulation and activation of ILC2s (63, 64). Studies with ILC2-deficient *Il7r* ^Cre^ *Rora* ^flox^ mice indicated that ILC2s are largely responsible for eosinophilia, mucous metaplasia and airway hyperresponsiveness in six-day-old mice infected with RV. Furthermore, in a model based on two time-shifted infections with heterologous RV strains, the ILC-dependent type 2 polarized immune response in infected premature mice resulted in a more severe disease outcome (65). Thus, similar to the conclusions derived from RSV infection models, these data indicate that lung ILC2 activation very early in life may significantly shape pulmonary immune responses later in life and could be related to an increased susceptibility for asthma development. Recently, the pandemic situation caused by the worldwide spread of the SARS-CoV2 virus has prompted a strong interest in respiratory virus research and immunomodulatory strategies to prevent and treat severe COVID-19 disease. Consistent with the strong epithelial damage observed in SARS-CoV2 lungs, global transcriptomic analyses of cells within the bronchoalveolar fluid indicated that the expression of IL-33 is upregulated in lungs of patients suffering from COVID-19 disease (66). In line with this, IL-33 blood concentrations were reported to be increased during active disease (67, 68). Moreover, the expression of IL-33 after stimulation of COVID-19 convalescent peripheral blood mononuclear cells with SARS-CoV2 peptides correlated with seropositivity for the viral spike glycoprotein (69) indicating that ILC2s or other ST2^+^ cells such as T cells, B cells and macrophages are functionally linked to the disease. Repeated intranasal treatment of mice with the protease allergen papain drives IL-33 release from the pulmonary epithelium and is a commonly used model to study the functions of lung ILC2s. Because the SARS-CoV2 genome encodes for the essential papain-like protease PLpro (70), Gomez-Cadena et al. administered this protein intranasally on five consecutive days to mice (71). Indeed, this treatment led to increased lung numbers of IL-5^+^ ILC2s and subsequent eosinophilia indicating that SARS-CoV2 could directly drive ILC2 responses in infected human lungs *via* PLpro. In the same study, flow cytometric analysis revealed a relative increase of ILC2 frequencies in the peripheral blood in mild to severe COVID-19 disease, while the pool of total ILCs was lower compared to healthy controls (71). Interestingly, within ILC2s, a subset expressing low levels of the receptor tyrosine kinase cKit was significantly expanded in patients with severe COVID-19 disease, possibly consistent with an accumulation of mature ILC2s. In addition to cKit, ILC2s of patients with severe COVID-19 disease displayed higher expression of the C-type lectin-like molecule NKG2D but lower expression of the classical ILC2 markers KLRG1 and CD25. An approach to stratify patients according to the median expression of NKG2D revealed that patients with low numbers of NKG2D^+^ ILC2s required more mechanical ventilation and longer hospitalization, albeit the numbers of analyzed patients was very low (71). Noteworthy, a further study found decreased frequencies of circulating ILC2s in severe but not moderate COVID‐19 disease along with an increased presence of damage markers, suggesting that low ILC2 numbers could be indicative of a more severe COVID-19 manifestation (72). Moreover, recent data suggest that increased serum concentrations of IL-13 are seemingly correlated to severe COVID-19 outcomes and IL-13 blockade reduced disease severity in a mouse model of SARS-CoV2 infection (73). However, it remains to be determined, whether ILC2s represent a significant source of this cytokine in COVID-19. Further experiments using *in vitro* studies with human lung cells, respiratory tissue of COVID-19 patients, rodent infection models and larger patient cohorts are required to prove the functional relevance of ILC2s in the pathogenesis of COVID-19 lung disease.

#### Bacterial Infections

Contrary to the rather intensively described functional roles of ILC2s during viral lung infections, studies linking ILC2s to bacterial infections of the lung are scarce. Saluzzo et al. were able to demonstrate that ILC2-deficient and IL-13-deficient mice showed a superior capacity to control *Streptococcus pneumoniae* infections (12). This observation was linked to activated ILC2s induced by postnatal upregulation of IL-33 expression in lung epithelial cells. By the production of IL-13, these ILC2s may shape the resident alveolar macrophages towards an anti-inflammatory M2-like phenotype promoting a quiescent steady-state immune environment, which, however, at the same time delays the immune response to *S. pneumoniae*. It was furthermore found that intratracheal IL-33 administration protected mice from systemic *Staphylococcus aureus* infection. This effect was dependent on the presence of lung ILC2s and the accumulation of eosinophils at the expense of pulmonary neutrophils (74).

#### Fungal Infections

Infections with fungal pathogens typically manifest at mucosal surfaces, particularly the lung, before they possibly spread systemically. It was demonstrated for many fungal pathogens that the formation of well-balanced immune responses is of critical importance for the host to be either resistant or susceptible (75). In recent years, changes in lung ILC2 numbers were described in the context of fungal infections or after exposure to fungal components. During *Aspergillus fumigatus* infection, increased pulmonary production of IL-25 was described, which resulted in the induction of an innate cell type secreting the cytokines IL-5 and IL-13 (76). In subsequent studies, fungal products such as the common cell wall constituent chitin or substances in extracts of the opportunistic pathogen *Alternaria alternata* were recognized as strong inducers of alarmin production and subsequent ILC2 activation after intranasal delivery (77, 78).

*Cryptococcus neoformans* is a fungal pathogen that can cause severe disease in immunocompromised individuals. Dominant lung Th1/Th17 immunity and the activation of classical macrophages are believed to ultimately facilitate fungal clearance, whereas highly polarized type 2 responses were observed to be rather detrimental. During the onset of pulmonary cryptococcosis, IL-33 expression is elevated and ILC2 numbers were strongly increased in lungs of these mice (79). Mice deficient in IL-33 signaling alone or in combination with IL-25 receptor deficiency developed less profound type 2 immunity in this model (80). In an intranasal infection model with a highly virulent *C. neoformans* strain, ILC2-deficiency in mice was associated with increased production of Th1 cytokines, elevated pulmonary frequencies of classically activated macrophages, improved fungal control and prolonged survival (79).

Overall, the here summarized data emphasize the significant role of ILC2s during viral lung infections, strongly suggest that ILC2s can orchestrate also pulmonary immune responses against fungal infections and even implicate a potential, yet not fully defined, involvement in bacterial lung infections.

## ILC2s as Drivers of Human Chronic Lung Diseases

Besides their before described crucial involvement in the immunological responses directed against lung tissue-affecting infectious pathogens, ILC2s also represent important cellular modulators of non-infectious chronic inflammatory and fibrotic diseases of the upper and lower airways. Here we will in particular discuss the disease-modifying influence of local ILC2 pools on the pathogenesis of asthma, cystic fibrosis and idiopathic pulmonary fibrosis, but reference should also be made to the role of these cells in the course of inflammatory disorders of the upper airway such as chronic rhinosinusitis with nasal polyps, which has been extensively summarized by other review articles (81, 82).

### ILC2s as Potential Drivers of Human Asthma

Asthma is a common inflammatory disorder of the airways typically characterized by airway hyperreactivity (AHR), mucus overproduction, IgE production and remodeling of the airways. It has been known for a long time that allergic asthma, the most common subtype, is characterized by the overproduction of the prototypical type 2 cytokines IL-4, IL-5 and IL-13 that promote the hallmark features of the disease (83). Consistent with a role of epithelial-derived alarmins as activators of type 2 cytokine production in the context of the disease, genome wide association studies identified the genes of IL-33 and its receptor IL1RL1 as highly replicated asthma susceptibility loci (84). In addition, several other studies found that IL-33, TSLP and IL-25 were upregulated on the RNA and protein level in lungs of patients with various forms of allergic asthma (85). Traditionally, allergic asthma was considered as a disease largely orchestrated by Th2 cells and their multifaceted interactions with other immune cells including IgE-producing B cells, eosinophils and mast cells. However, with the discovery of ILC2s as a novel lymphocyte subset with large functional similarities and profound pro-asthmatic roles in asthma models, e.g. induced by intranasal administration of plant proteases, house dust mites, fungal extracts or alarmins, this concept was recently revisited. Accordingly, the functional contribution of ILC2s to the development of human asthma is a subject of extensive investigation. Indeed, the frequencies and the activation status of ILC2s, which are in most studies distinguished from other ILCs by their expression of CRTH2, have been shown to be increased in blood, bronchoalveolar lavage and lung tissue of patients with asthma (86–88). Here ILC2 frequencies were particularly increased in eosinophilic asthma compared with non-eosinophilic asthma and ILC2 numbers positively correlated with surges in eosinophils and M2-like macrophages (89, 90). Seemingly, women with severe asthma have an even higher increase in the numbers of circulating ILC2s compared to men, likely due to sex hormone-mediated ILC2 suppression (91). Consistent with their role as drivers of asthma symptoms, ILC2s were increased during the allergy season (92) and their local numbers in the lung increased significantly after segmented allergen challenge and went along with an increased expression of genes related to type 2 immunity (93). Noteworthy, it was also found in the latter study that systemic ILC2 frequencies decreased after allergen challenge indicating that ILC2s can be actively recruited from the blood. Similarly, ILC2s increased in the lungs of patients with moderate allergic asthma after allergen inhalation challenge albeit the increase was transient (94). Importantly, further indirect evidence for potential pathogenic roles of ILC2s in asthma was generated in studies reporting decreased ILC2 frequencies after successful therapeutic interventions (95, 96). Because loss of epithelial barrier integrity is common in asthmatic lungs, Sugita et al. performed *in vitro* co-culture experiments with human bronchial epithelial cells and ILC2s and *in vivo* experiments in mice to study direct interactions of ILC2s with epithelial cells (97). Overall, these data indicated that ILC2s, mainly through production of IL-13, were potent inducers of bronchial epithelial barrier leakiness, although further studies are needed to determine whether a similar mechanism is functional in the human pulmonary environment. Finally, a recent study demonstrated a role of human KLRG1^–^ ILC2s in reducing grass-pollen allergy. This novel subtype of ILC2s produces immunomodulatory IL-10, was reduced in allergic patients, but notably was restored in such patients receiving grass-pollen sublingual immunotherapy (98).

Collectively, an increasing number of studies in human asthma supports the notion that alarmin-dependent ILC2 activation is a major driver of the disease. However, further studies elucidating their relative disease-promoting importance compared to Th2 cells and identifying specific ways for their targeted modulation are urgently needed.

### Role of ILC2s in Human Fibrotic Lung Diseases

Fibrosis is characterized by excessive tissue accumulation of extracellular matrix components, resulting from chronic inflammation and dysregulated tissue repair. The pathogenesis of fibrosis depends on complex mutual interactions between different immune cell subpopulations, mesenchymal and parenchymal cells. Given the key pathogenic roles of type 2 cytokines in experimental models of fibrotic diseases, studies recently addressed the specific role of ILC2s for fibrosis development in the human lung (15, 99). Pulmonary fibrosis is a feature of several lung diseases including cystic fibrosis (CF), idiopathic pulmonary fibrosis (IPF) and also chronic allergic diseases. Although, the contribution of ILC2s in pulmonary fibrosis is supported by several data from animal models, their precise role in humans has been addressed only in few studies and thus remains largely unclear.

In bronchoalveolar lavage fluid and sputum of patients with CF, the presence of IL-33 as well as IL-5, IL-9 and IL-13 was upregulated, suggesting that local type 2 responses may somehow contribute to CF pathogenesis (100–102). In line with this, the risk for manifestation of allergic asthma was reported to be higher in patients with CF (103). Morelli et al. reported an association of a single nucleotide polymorphism in the IL-9 gene with high *Aspergillus*-specific IgE levels in females with CF (101). Based on additional studies in mice, demonstrating that ILC2-derived IL-9 triggers CF-associated inflammation *via* a complex vicious cycle that includes activation of mast cells, it was speculated that a similar mechanism could support the infection-dependent disease exacerbation in CF. Moreover, decreased numbers of CCR6^+^ ILC2s in peripheral blood of CF significantly correlated with advanced pulmonary failure (104), suggesting a pathophysiologically relevant alteration of the ILC2 migration patterns in patients with severe CF. In patients with IPF, increased levels of IL-25 and also ILC2s were observed. Most recently, ILC2 frequencies in IPF were shown to correlate negatively with Regnase-1 expression levels, a factor important for posttranscriptional processes in immune cells. In addition, a high number of ILC2s was associated with poor IPF prognosis (105). Because a similar functional mechanism was described also in lungs of Regnase-1 deficient mice subjected to bleomycin-treatment, these data suggested that dysregulated ILC2s may accelerate the progression of IPF.

## Numeric Regulation of Local ILC2 Pools at Inflamed or Fibrotic Pulmonary Tissue Sites by ILC2 Recruitment and Trafficking

### Context-Dependent Tissue Homing and Interorgan Trafficking of ILCs and ILC Precursors

As already discussed in the previous chapters, based on important pioneering experiments in parabiotic mice with a joint blood circulation, ILCs have long been assumed to represent strictly organ resident immune cells, whose maintenance in secondary lymphoid and non-lymphoid organs in the adult organism is guaranteed by local expansion and self-renewal (7). However, the life-long existence of ILCs and precursor ILCs in the peripheral blood in humans (9, 106–108), altered frequencies of circulating ILCs under inflammatory conditions (72, 104, 109–111), an at least moderate infiltration of donor-derived ILCs in mesenteric lymph nodes, lung and intestinal tissue even in the parabiotic mouse model under chronic inflammatory conditions (7) and the observation that IL-25-induced inflammatory ILC2s (iILC2s) behave like circulating cells, strongly argue for a maintained and context-dependent capacity of ILCs to traffic between different organs (112). In accordance with this concept, Ai Ing Lim et al. successfully established the model of tissue ILC differentiation, which is based on the identification of circulating ILC precursors in the peripheral blood. Once infiltrated into peripheral tissue, these ILC precursors are able to undergo differentiation into all helper ILC subsets triggered by local environmental signals (107). In this context, it is also interesting to note that results from several studies indicated that the analogy between innate helper ILCs and the adoptive T helper cell compartment is not exclusively limited to their cytokine, transcription factor and effector function profiles but also concerns the expression of tissue homing receptors (e.g. S1PR1, CCR9, CCR4, LFA-1, α4β7 integrins) and specific aspects of their migratory behavior (19, 109, 110, 113–115). Indeed, *in vivo* and *ex vivo* experimental studies clearly demonstrated that both T cells and ILCs use CCR7 and S1PR1 for reaching and egressing secondary lymphoid organs, respectively (113, 114, 116, 117). Being especially well studied in ILC1s and ILC3s, it has been documented that these ILC subpopulations are obviously able to undergo an adaption of their tissue homing receptor expression profile once they have entered into secondary lymphoid organs, thus predisposing them for trafficking towards specific non-lymphoid organs, like the skin or gut (113, 118, 119). In contrast, the homing receptor profile of ILC2s and precursor ILC2s appeared to be predetermined already in the bone marrow and ILC2s are thus able to bypass secondary lymphoid organs and instead traffic directly from the bone marrow towards the peripheral target organ (113, 118). Besides the peripheral distribution of bone marrow-egressed ILCs and precursor ILCs, several studies also described the migration of mature ILCs between different peripheral non-lymphoid organs (19, 110). A landmark study published by Yuefeng Huang et al. convincingly demonstrated that for instance lung infiltrating IL-25-induced iILC2s originated from ILC2s that naturally reside in the intestinal mucosa (19). In a similar way, data acquired in a cohort of patients diagnosed for ankylosing spondylitis implicated the existence of an ILC3 homing axis between the gut and the inflamed joints, which obviously allows a targeted distribution of gut-derived and disease-modulating α4β7-expressing ILC3s (110).

In addition to determining the interorgan trafficking of ILCs, the integrin and chemokine receptor expression profile turned out to predispose specific ILC subsets for the accumulation within a particular anatomical niche of the target organ. For instance, in analogy to CD8 Trm cells, intraepithelial NKp44^+^CD103^+^ ILC1s could be characterized by an increased surface expression of the β7 integrin, which together with CD103 forms the αEβ7 heterodimer as a potent binding partner for epithelial E-cadherin and, thus, a marker for epithelial retention (120).

### Regulation of ILC2 Trafficking in the Context of Inflammatory Lung Diseases

**Figure 1:**
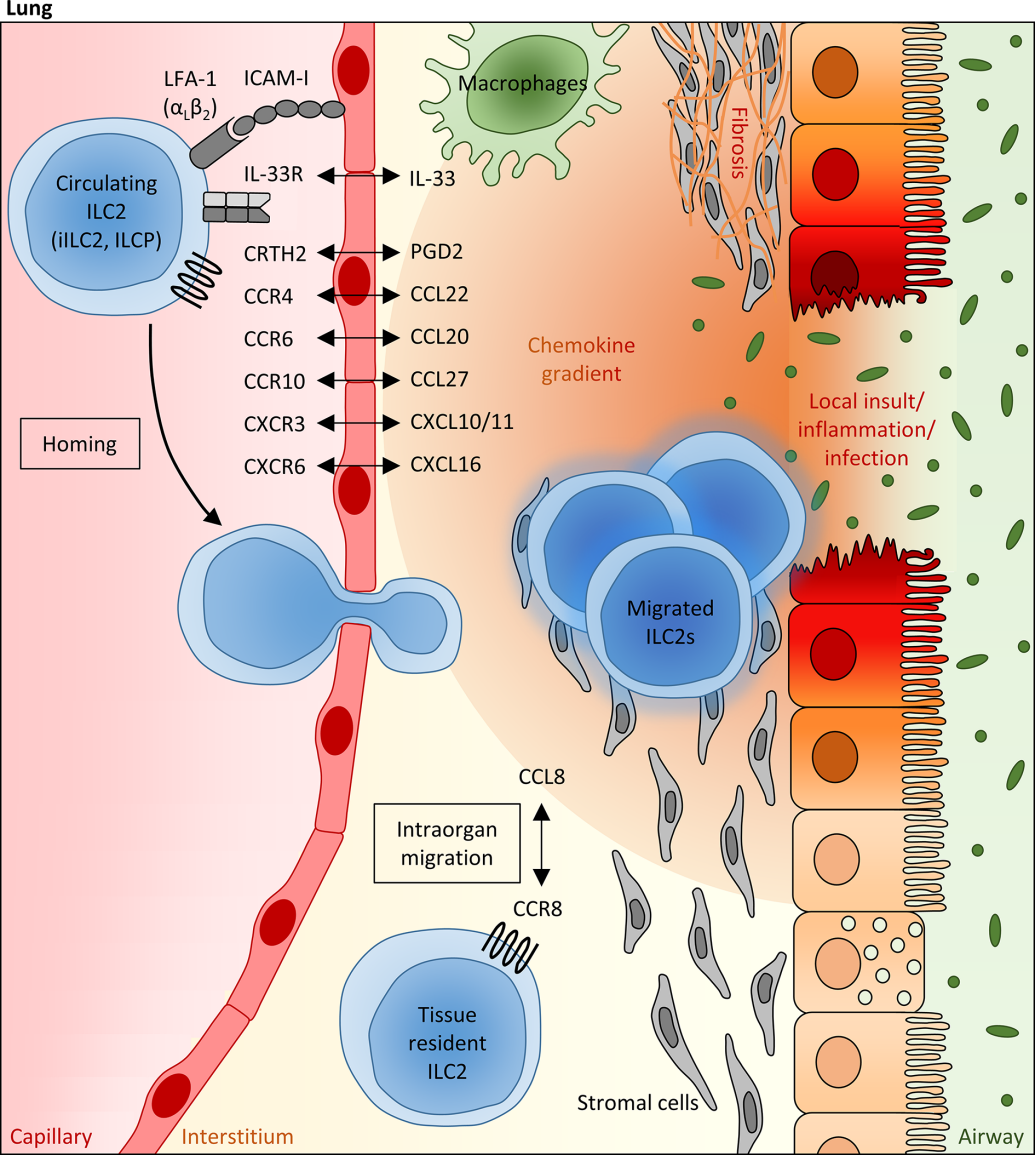
Mechanisms regulating the pulmonary recruitment and spatial distribution of ILC2s during adulthood. Infectious or allergic lung inflammation and fibrosis are able to trigger the pulmonary recruitment of circulating blood ILC2s, including precursor ILCs (ILCP) egressed from the bone marrow, which is at least partly mediated *via* IL-33, as well as mature “inflammatory” ILC2s (iILC2s) primed in the gut. In this scenario, the local pulmonary milieu has been attributed to a crucial modulating function, which can influence the migratory behavior of systemic ILC2s and their intraorgan distribution within the lung tissue. In particular, lipid mediators (PGD2), cytokines (e.g. IL-33) and chemokines (mainly CCL20, CCL22, CCL27, CXCL1, CXCL10 and CXCL16), whose local expression levels are markedly altered under inflammatory conditions and which can be largely attributed to epithelial and stromal cells as well as innate immune cells (representatively shown as macrophages), are assumed to function as potent numeric regulators of pulmonary ILC2 pools, allowing the adaption to local requirements. Besides the chemokine receptor profile of ILC2s, the presence of specific integrins (e.g. LFA-1) on their surface influences the lung homing capacity of circulating ILC2s. Within the lung, the CCR8/CCL8 axis has been identified as a crucial regulator of the spatial distribution of pulmonary ILC2s, promoting their peribronchial enrichment in a niche defined by stromal cells.

As described before, there is continuously increasing experimental evidence for the concept that tissue migrating ILCs together with the enforced proliferation of organ resident ILCs contribute to the inflammation-triggered expansion and modulation of local ILC pools (16, 93, 107). However, the existing data strongly implicate that the functional and numeric relevance of newly recruited ILCs for local immune responses as well as the pattern of involved chemokines and adhesion molecules depend on the respective target organ, the pathological context and on the ILC subtype (7, 112, 121, 122). As ILC2s have been established as the predominant helper ILC population in the context of allergic and inflammatory lung diseases (9), we will here describe in detail mediators and signaling pathways regulating their lung homing capacity and pulmonary recruitment upon inflammatory conditions (**Figure 1**), while the mechanisms underlying the inflammation-triggered expansion of lung resident ILC2s in the human and murine system have been reviewed elsewhere (16, 123). As alarmin-like cytokines (e.g. IL-25, IL-33, TSLP) are very potent inducers of ILC2 proliferation, survival and activation (16, 32) and are released by damaged lung epithelial cells in direct proximity to lung resident ILC2s as a very early response to exogenous or endogenous inflammatory triggers, it appears likely that expansion of local ILC2 pools is of predominant importance during initial phases of acute inflammatory disorders, while the relevance of pulmonary ILC2 recruitment might increase with the subsequent development of an inflammatory milieu (including increased expression of chemokines). Moreover, further arguing for a slightly delayed involvement of newly recruited ILC2s, most blood-derived ILCs enter their peripheral target tissue as precursor ILCs, which still have to undergo final differentiation induced by the local inflammatory micromilieu (107). However, it will be important to define the pathogenic relevance of lung ILC2 recruitment versus expansion of local ILC2 pools more precisely for specific pulmonary diseases in future studies.

### IL-33-Mediated Pulmonary Recruitment of Bone Marrow-Derived ILC2s

Experimental induction of allergic lung inflammation was found to result in a decreased ILC2 frequency in the bone marrow, while the number of pulmonary ILC2s increased subsequently, strongly implicating a targeted ILC2 migration from the bone marrow towards the inflamed tissue site (124). In this context, it was interesting to learn that the IL-1 family member IL-33, whose serum levels are markedly elevated in asthma patients (125), does not only function as a key activator of ILC2s in peripheral organs, but also crucially impacts on the inflammation-triggered egress of ILC2s from the bone marrow and their subsequent lung-directed hematogenous migration (126). Indeed, it could be demonstrated *in vivo* that the intravenous delivery of recombinant IL-33 into wild type mice resulted in a decreased total number of ILC2 precursor cells in the bone marrow, while the *de novo* generation of ILC2 precursor cells in the bone marrow turned out to be independent from IL-33 signaling (126). Regarding the underlying mechanism, the IL-33-induced downregulation of the bone marrow retentive chemokine receptor CXCR4 has been identified as a relevant factor for the bone marrow egress of ILC2 precursor cells at least during the early postnatal phase (126). Moreover, performed *in vivo* and *in vitro* studies based on the use of neutralizing antibodies directed against specific integrins were able to demonstrate that the bone marrow-to-lung route depends on the binding of the ILC2-expressed integrin heterodimer LFA-1 to the endothelial adhesion molecule ICAM-I (124).

### Role of Lipid Mediators and Chemokines for the Lung Migration of Circulating Blood ILC2s

Focusing on the subsequent pulmonary recruitment of bone marrow-egressed and systemically circulating blood ILC2s, an elegant clinical study in a small cohort of patients with mild to moderate asthma was able to describe that allergen challenge resulted in a simultaneous but opposite regulation of ILC2 counts in the peripheral blood and lung tissue: While the number of peripheral blood ILC2s significantly decreased 24 hours after antigen provocation, there was a parallel enrichment of ILC2s in the bronchoalveolar lavage (93). A similar decrease of ILC2 counts in the peripheral blood was also described in patients infected with tuberculosis, although here this phenomenon was not restricted to ILC2s, but also included circulating ILC1s and ILC3s (111). In accordance with these observations in the human system, intravenously transferred murine ILC2s were found to successfully migrate to the alveoli in a mouse model of cytokine-induced lung inflammation, while this was not the case in the absence of inflammatory triggers (28, 31). Thus, these *in vivo* acquired data strongly supported the concept that local ILC2 pools in the lung are replenished by hematogenous ILC2s in the presence of inflammatory stimuli. Taking into account the impact of the local inflammatory milieu on this phenomenon, the authors of the aforementioned clinical study interestingly observed an inverse correlation between the number of ILC2s remaining in the blood circulation and the levels of the mast cell-released lipid mediator PGD2 and the chemokine CXCL12 in the bronchoalveolar lavage of allergen challenged patients, whereby both molecules could be characterized as potent inducers of ILC2 chemotaxis (36, 93). It is also worth mentioning here that the leukotriene E4 (LTE4), which represents a biomarker in asthma (127) and whose receptor CysLT_1_ is present on the surface of a relevant fraction of circulating ILC2s, was able to mediate additive effects on the PGD2-induced ILC2 migration in an *in vitro* chemotaxis assay (40). Besides LTE4, the family of CysLT leukotrienes also includes LTC4 and LTD4, whereby LTC4 represents the parent CysLT leukotriene and is converted to LTD4 and LTE4 after being released to the extracellular compartment. Indeed, also LTC4 and LTD4 were found to induce chemotaxis of ILC2s *in vitro*, although to a lower extend than LTE4, and LTC4 could be identified as a potentiator of IL-33-induced lung inflammation (40). Accordingly, mice challenged with a combination of LTC4 plus IL-33 showed a significantly increased pulmonary accumulation of ILC2s (128). However, it remains open, whether the observed phenomenon might at least partly be due to a potential influence of LTC4 on the *in vivo* lung recruitment of ILC2s or mainly depends on the described capacity of LTC4 to trigger IL-33-mediated proliferation of local ILC2 pools (128). In contrast to CysLT leukotrienes, the leukotriene B4 (LTB4) seems to impact on the number and activation status of lung ILC2s predominantly *via* indirect and IL-33-dependent mechanisms, although a minor direct influence of LTB4 on ILC2 chemotaxis could at least be demonstrated *in vitro* (129).

In view of the broadly established general role of chemokine signaling for lung homing of different lymphoid immune cell populations (130, 131), it appears worth to pay more detailed attention to the chemokine receptor repertoire of ILC2s in the context of pulmonary inflammation. Even under steady-state conditions, the vast majority of peripheral blood ILC2s show a relevant surface expression of the chemokine receptor CCR4, whose ligand CCL22 represents a well-established Th2 cell attractant and was found to potently trigger *in vitro* ILC2 migration (26, 132, 133). It is thus strongly assumed that locally enhanced levels of CCL22 in the respiratory tract, as for instance observed in patients diagnosed for idiopathic pulmonary fibrosis or allergic rhinitis and in experimental asthma models (134–136), are able to promote pulmonary ILC2 recruitment. Accordingly, intravenously transferred CCR4-deficient ILC2s failed to migrate into the lung of mice intranasally challenged with the protease allergen papain (26). As an interesting side note, it should be mentioned that CCR8, which also represents a Th2-associated chemokine receptor and is closely related to CCR4, seemed to be dispensable for the lung migration of ILC2s in the same experimental *in vivo* system despite its relevant expression in pulmonary ILC2s (26). Besides a marked surface expression of CCR4, circulating ILC2s in the peripheral blood often express the chemokine receptor CCR6 (9, 109), which turned out to be of particular relevance for the lung homing capacity of this helper ILC subpopulation in the context of lung inflammation (104). Accordingly, adult patients suffering from CF could be characterized by a decreased frequency of circulating CCR6^+^ ILC2s in the peripheral blood and this phenomenon interestingly correlated with the severity of respiratory dysfunction, strongly implicating a CCR6-mediated migration of systemic ILC2s into the inflamed lung tissue of these patients (104). Interestingly, a similar decrease of peripheral blood CCR6^+^ ILC2s and a significant inverse correlation between the number of circulating ILC2s and serum levels of the CCR6 ligand CCL20 have recently been described in patients suffering from COVID-19 disease (72), further pointing to the involvement of CCR6/CCL20-initiated signaling cascades in the inflammation-triggered lung recruitment of ILC2s. The altered frequency of CCR6^+^ ILC2s in the peripheral blood of COVID-19 patients turned out to be accompanied by a significant reduction of the overall small population of CXCR3^+^ ILC2s. Together with the observation that high serum concentrations of CXCL10 and CXCL11 (CXCR3 ligands) associated with decreased CXCR3^+^ ILC frequencies in the blood of these patients, it appears likely that also CXCR3-ligand-dependent effects are able to impact on the pulmonary enrichment of blood-derived ILC2s in the context of viral lung infections (72). Analyses in murine asthma models further point to CXCR6 as an additional chemokine receptor with direct chemoattractant effects on systemic ILC2s (137, 138). Indeed, the CXCR6 ligand CXCL16 is constitutively expressed by human lung epithelial cells and alveolar macrophages (139, 140) and even increased CXCL16 levels have been detected in OVA- or IL-33-challenged mice (137). In accordance with the *in vitro* demonstrated capacity of recombinant CXCL16 to induce chemotaxis of lung tissue-derived murine ILC2s, antibody-mediated *in vivo* neutralization of CXCL16 was able to successfully dampen the pulmonary accumulation of ILC2s in the context of experimentally induced asthma (137). Although classically described as a skin homing receptor, the chemokine receptor CCR10 was found to be expressed on the surface of more than one third of pulmonary ILC2s in non-inflamed human lungs and, moreover, asthma patients could be characterized by a markedly increased frequency of CCR10^+^ ILC2s and elevated levels of CCL27 in the peripheral blood, implicating a potential impact of CCR10 and its ligands CCL27 on the inflammation-dependent enrichment of pulmonary ILC2 pools. In accordance with this hypothesis, *in vivo* blockade of CCR10 ligands indeed abolished the pulmonary presence of CCR10^+^ ILC2s in allergen-exposed mice (141). Taking into account the described capacity of CCR10^+^ ILC2s to ameliorate allergic lung inflammation, the CCR10-mediated recruitment of pulmonary ILC2s might be of particular relevance for maintenance or restoration of the balance between the pro-inflammatory effects of lung ILC2s and their capacity to enhance resolution of inflammation.

### Intrapulmonary Distribution of ILC2s

Notably, local inflammatory conditions in the lung turned out to not exclusively impact on the recruitment and tissue migration of systemic ILC2s or precursor ILCs, but apparently also influenced their spatial distribution within the inflamed pulmonary tissue (**Figure 1**). Indeed, accumulation of ILC2s in the peribronchial and perivascular space could be demonstrated in murine lungs after intranasal administration of recombinant IL-33, which was obviously dependent on the CCL8-CCR8 ligand-receptor interaction (28). Upregulation of the chemokine receptor CCR8 has been described upon IL-33 exposure in lung-infiltrating ILC2s and turned out to be accompanied by increasing pulmonary levels of CCL8, as the respective ligand. Indicating functional evidence, antibody-mediated *in vivo* blockade of CCR8 was able to significantly dampen the inflammation-induced accumulation of ILC2s in peribronchial tissue areas (28). At first glance, these data seem to contradict the before described dispensability of CCR8 for the pulmonary recruitment of ILC2s from the peripheral blood circulation and the inefficacy of the CCR8 ligand CCL8 to promote *in vitro* migration of ILC2s (26). However, this seeming controversy might most likely be explained by the fact that ILC2s in blood and lung tissue are surrounded by different microenvironments and the functional outcome of the CCR8/CCL8 axis for ILC2s might thus be critically influenced by additional, yet to be defined, co-factors. In addition to chemokine signaling, the perivascular presence of adventitial stromal cells has been identified as another factor, which relevantly promotes the spatial accumulation of pulmonary ILC2s around lung arteries upon inflammatory conditions (13, 138). Involving adventitial stromal cell-derived IL-33 and TSLP as well as ILC2-derived IL-13, the bi-directional adventitial stromal cell-ILC2 crosstalk was found to be triggered by inflammatory stimuli and to generate an optimized niche for enhanced ILC2 enrichment and activation (13).

## Discussion of Clinical Implications

In view of the increasingly perceived relevance of interorgan trafficking of non- or not yet organ resident ILCs and precursor ILCs, the cytokine- and chemokine-driven recruitment of this innate cell population towards local inflammatory tissue sites has drawn our attention also with regard to resulting therapeutic implications for allergic or inflammatory diseases. In the following section, we will thus discuss the therapeutic potential and targetability of selected signaling cascades with a described relevance for pulmonary ILC2 recruitment in patients suffering from allergic, inflammatory or fibrotic lung diseases. In this context, it is obvious to mention the successful development of the fully human antibody REGN3500, which specifically recognizes human IL-33, is able to efficiently block the IL-33 downstream signaling and is currently under clinical development for the treatment of asthma and COPD (142). In addition to REGN3500, etokimab represents a second clinically applicable anti-IL-33 antibody, whose suppressive *in vivo* effect on the function of IL-33 has successfully been validated in phase 1 and phase 2a clinical trials (143). As the alarmin IL-33 represents a well-established promotor of the bone marrow-to-lung recruitment of ILC2s under inflammatory conditions and also serves as a key activating cytokine for pulmonary ILC2 pools (125, 126), its antibody-mediated blockade can be expected to relevantly dampen the local involvement of ILC2s in the induction or maintenance of lung inflammation. Due to the rather broad expression profile of the IL-33 receptor ST2 on different immune cell subtypes, targeting IL-33 signaling does not represent an ILC2-specific strategy and can more be seen as a global approach to efficiently block type 2 immunity, including also marked inhibitory effects on lung infiltrating Th2 cells, eosinophils, mast cells and macrophages (144–148). Besides the advanced clinical validation of blocking IL-33 antibodies in inflammatory lung diseases, it will thus be exciting to define in how far the assumed therapeutic efficacy of anti-IL-33 antibodies depends on their capacity to interfere with the activation and recruitment of pulmonary ILC2s.

Due to the local upregulation of CCL22 in inflamed and/or fibrotic lung tissue, for instance in patients diagnosed for asthma or idiopathic pulmonary fibrosis (149, 150), its proven capacity to chemoattract ILC2s (26, 132) and the presence of the corresponding chemokine receptor CCR4 on the vast majority of circulating ILC2s (109), the CCL22/CCR4 axis emerged as an attractive target for controlling the additional pulmonary recruitment of peripheral ILC2s in the course of inflammatory lung diseases. Interestingly, molecular docking studies indicated that the anti-malarial hydroxychloroquine, whose potential benefit for patients suffering from COVID-19 disease had been under intensive debate (151), was able to interact with the active site of CCR4, implicating that an inhibitory effect on CCR4 signaling might, at least partly, underly the reported therapeutic and immunomodulatory capacity of this drug in the clinical context of asthma (152, 153). However, despite the availability of the humanized anti-CCR4 antibody mogamulizumab, which has been clinically approved for the treatment of adult T cell lymphoma (154, 155), till today, there exist no clinical or preclinical *in vivo* data confirming a clinical benefit of antibody-mediated CCR4 blockade in the therapeutic context of allergic or inflammatory lung disease. Indeed, intravenous injection of a blocking anti-CCR4 antibody into guinea pigs previously sensitized with OVA, was not able to relevantly decrease the inflammatory cell infiltrates and chemokine levels in the lung after local antigen challenge (156). It might be assumed that therapeutic strategies based on the use of CCL22-neuralizing antibodies or small molecules are superior to a direct inhibition of CCR4, because they would imply the feasibility and potential efficacy of a topic inhalative drug application and also prevent partial agonistic or inverse agonistic activities, which might otherwise be associated with the use of receptor antagonists (157, 158). Indeed, the CCL22-neutralizing decoy molecule GPN136 was able to successfully decrease the level of lung inflammation in an experimental *in vivo* model of allergic asthma. However, considering that CCR4 is expressed on various immune cell populations besides ILC2s, such as Th2 cells, dendritic cells and eosinophils (133), it is difficult to estimate in how far this GPN136-mediated therapeutic effect depends on a particular blockade of the pulmonary ILC2 recruitment.

In the field of classic inflammatory lung pathologies, therapeutic inhibition of CCR4 signaling has mainly been discussed as a potential treatment strategy for allergic asthma so far, while the interplay between CCR6 and its ligand CCL20 recently arose as another promising target for a numeric regulation of local ILC2 pools in the inflamed lung tissue, which might be of particular relevance for the clinical management of CF. Indeed, significantly increased levels of CCL20 could be detected in the bronchoalveolar lavage of CF patients (159) and advanced pulmonary failure in this disease turned out to be accompanied by decreased frequencies of systemically circulating CCR6-expressing ILC2s (104). Moreover, based on preclinical analyses in a humanized mouse model, blood-derived human ILC2s could be identified as potent inducers of eosinophil and neutrophil accumulation in the lung and relevantly impacted on the pulmonary tissue structure (104). Taken together, these data strongly implicated that increased CCL20/CCR6-driven ILC2 recruitment from the blood towards the inflamed lung tissue promotes the clinical exacerbation of CF (104), making it an attractive future research objective to experimentally investigate the therapeutic capacity of specific and already available pharmacological inhibitors of the CCL20-CCR6 axis, such as anti-CCR6 or anti-CCL20 antibodies or small molecular inhibitors (160), in this lung-affecting disease. However, following this concept, it is inevitable to take into account the relevant expression of CCR6 also on adaptive immune cells (mainly Th17 and Treg cells, but also γδ T cells and B cells) and the resulting and intensively described influence of the CCL20-dependent immune cell recruitment on the immunological tissue homeostasis of various organ systems besides the lung, such as the gastrointestinal tract and the nervous system (160–163). Although the development of topic lung-selective administration routes (e.g. *via* inhalation) might potentially allow to minimize systemic effects, experimental data acquired in several *in vivo* model systems strongly suggest that therapeutic blockade of the CCL20/CCR6-mediated pulmonary immune cell recruitment might not exclusively decrease the local enrichment of ILC2s (72, 104), but will also relevantly impact on the Th17/Treg cell ratio in the lung (160, 163, 164) and thereby potentially target another important aspect in the immunopathogenesis of CF, which is a pathological predominance of Th17 cells (165, 166). At the current state, all this remains speculative and further studies are needed to preclinically validate the therapeutic efficacy of CCL20/CCR6 inhibitors in CF and to define the particular relevance of CCR6-expressing blood-derived ILC2s in this context.

Regardless of whether one considers IL-33/ST2, CCL22/CCR4 or CCL20/CCR6 as target structure for a potential therapeutic modulation of the lung recruitment and local activation of ILC2s, it becomes obvious that none of these strategies will allow an exclusive interference with this innate cell population. In general, the main unsolved obstacle in realizing the idea of ILC-based therapeutic concepts is that fully ILC-specific target structures are still lacking. This phenomenon can mainly be explained by the broad analogy between ILCs and Th cells, which encompasses their responsiveness to key activators of effector function and chemotaxis (16). As a highly innovative future attempt, strategies implementing cellular therapy and thus the adoptive transfer of *ex vivo* expanded and potentially pre-selected ILC populations might be able to overcome this limitation and allow to exclusive modulate the ILC compartment. Further following this hypothetical idea, CCR10^+^ ILC2s can be considered as interesting candidates for cellular therapy of patients suffering from severe asthma. Based on the combined interpretation of descriptive human data acquired in blood- and lung tissue-derived ILC2s and functional murine analyses in an experimental model of intranasal allergen-challenge, a suppressive function of lung infiltrating CCR10^+^ ILC2s on allergic airway inflammation has been strongly suggested, which was obviously due to ILC1-like properties of this specific ILC2 subfraction and their increased capacity for IFNγ secretion (141). Provided that it will be feasible to *ex vivo* expand CCR10^+^ ILC2s under the stable maintenance of their functional properties, it can thus be assumed that asthma patients might benefit from the adoptive transfer and the subsequent intended pulmonary enrichment of these suppressive ILC2s. In this context, it is worth mentioning that human CCR10^+^ ILC2s also differed from their CCR10-negative counterparts with regard to several other surface markers and could for instance be characterized by a decreased expression of the inhibitory molecules CTLA‐4 and PD‐L1. This makes it interesting to compare different subfractions within the CCR10^+^ ILC2 population on a functional level and, thereby, potentially further narrow down the surface marker profile indicative for high suppressive and anti-allergic activity. Very recently, IL-10-producing KLRG1^+^ ILC2s emerged as other hypothetically interesting candidates for therapeutic cell transfer in the context of allergic asthma therapy (98). In particular the observation that patients with allergy showed a decreased frequency of this Th responses-attenuating ILC2 subpopulation and that the induction of IL-10-secreting ILC2s in these patients by allergen immunotherapy was associated with clinical response (98) strongly implicated that targeted enrichment of IL10^+^KLRG1^+^ ILC2s could be an attractive, albeit technically challenging therapeutic target to strive for in the future clinical management of asthma patients.

## Author Contributions

All authors listed have made a substantial, direct, and intellectual contribution to the work and approved it for publication.

## Funding

This work has received funding from the DFG; German Research Foundation (TRR241: A07, A03, C04; FOR2886 TP1).

## Conflict of Interest

MFN has served as an advisor for Pentax, Giuliani, MSD, Abbvie, Janssen, Takeda and Boehringer.

The remaining authors declare that the research was conducted in the absence of any commercial or financial relationships that could be construed as a potential conflict of interest.
